# Supplementary material for: Shadowing and shielding: Effective heuristics for continuous influence maximisation in the voting dynamics
Source: PLoS One. 2021 Jun 18;16(6):e0252515. doi: 10.1371/journal.pone.0252515 (PMC8213046; doi:10.1371/journal.pone.0252515)
Supplement: S1 Appendix — (PDF) [file pone.0252515.s001.pdf]

Supplementary material to the paper: Shadowing and shielding: effective heuristics for continuous influence maximisation in the voting dynamics

Guillermo Romero Moreno[1,*], Sukankana Chakraborty[1], and Markus Brede[1]

[1]University of Southampton, Electronics and Computer Science, Southampton, SO171BJ, United Kingdom
[*]Guillermo.RomeroMoreno@soton.ac.uk

ABSTRACT

This document comprises the supplementary materials for the paper *Shadowing and shielding: effective heuristics for continuous influence maximisation in the voting dynamics*. The additions to the paper presented here are as follows.

Contents of the supplementary material

- Section 1: Details of the network employed in the main manuscript
- Section 2: Distribution of optimal allocations for other different number of nodes targeted by the opponent
- Section 3: Full derivations of the Taylor expansions in the limit of low external influence
- Section 4: Full derivations of the Taylor expansions in the limit of large external influence
- Section 5: Numerical results related to the exploitability of a passive controller depending on the degree of the nodes mistargeted
- Section 6: Comparison of analytical and numerical results in the continuous regime when in budget disadvantage
- Section 7: Dependence of optimal influence allocations in the continuous regime on node degree
- Section 8: Influence maximisation in the discrete regime
- Section 9: Proof of uniqueness of Nash Equilibrium
- Section 10: Numerical results about the convergence to the Nash equilibrum
- Section 11: Extension to other network topologies
- Section 12: Proof of concavity
- Section 13: Iterations to optimal IM of the proposed heuristics
- Fig 1: Histogram of the degree distribution of the network employed
- Fig 2: Distribution of optimal influence allocations for other values of K nodes targeted by the opponent
- Fig 3: Numerical results for exploitability of a passive controller when nodes of certain degree are misallocated
- Fig 4: Comparison of analytical and numerical results in the continuous regime when in budget disadvantage
- Fig 5: Dependence of optimal influence allocations on node degree
- Fig 6: Numerical results of the effect of shadowing, shielding and node degree on optimal allocations in the discrete regime

- Fig 7: Numerical results of convergence to the Nash equilibrium through iterative gradient ascent
- Fig 8: Distribution of optimal influence allocations for various network topologies
- Fig 9: Percentage enhancement of control by optimal continuous allocations over optimal discrete allocations for various network topologies
- Fig 10: Average optimal allocations given to groups related to shadowing and shielding in various network topologies
- Fig 11: Numerical results of correlations between targeting strength and node degree for various network topologies
- Fig 12: Comparison of various heuristics to optimal allocations for various network topologies
- Fig 13: Comparison of various heuristics to optimal allocations for other real–world network topologies
- Fig 14: Alternative assessment of the proposed heuristics
- Table 1: Network properties of the network topologies tested

1 Details of the network employed

For all experiments in the main manuscript, we have used an email interaction network[1] as an example of a common topology found in social networks. This heterogeneous network is unweighted and undirected, with a unique component of size $N = 1133$, mean degree $\langle d \rangle = 9.62$, and degree assortativity $\delta = 0.078$. Its degree distribution can be seen in Fig 1. Experiments on other network topologies are shown below.

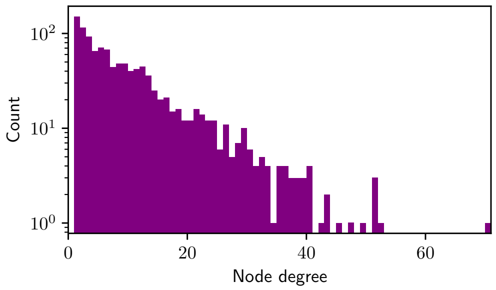

Figure 1. Degree distribution of the studied network.

2 Distribution of optimal allocations for other different number of nodes targeted by the opponent

In this section, we explore the distribution of optimal allocation distribution as shown in Fig 1a from the main manuscript, but for other values of K nodes targeted by the opponent. Namely, we include the case of $K = 8$ (left), $K = 16$ (middle), and $K = 32$ (right) nodes. As can be seen from the figure, the allocation distribution smoothens as K increases. This effect is indirectly captured in Fig 1b of the main manuscript, as entropies of the allocation distribution decrease with K.

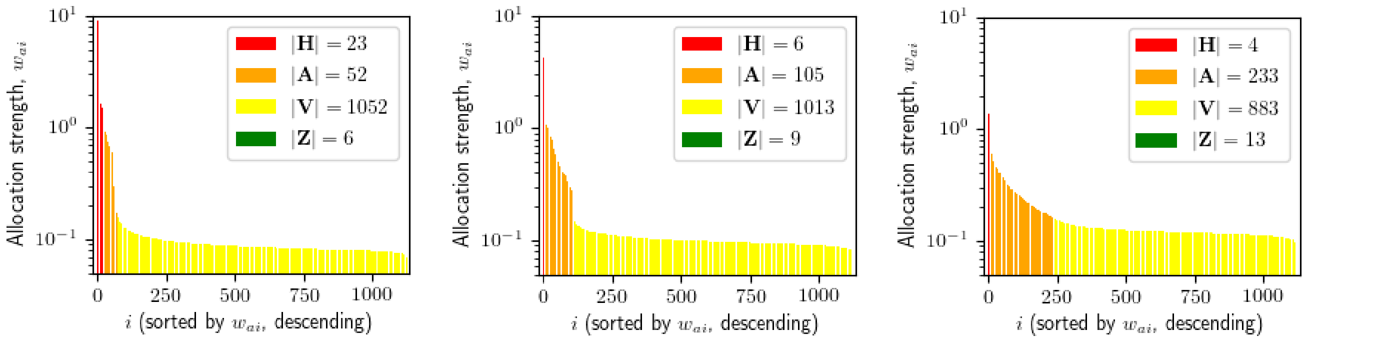

Figure 2. Distribution of optimal influence allocations sorted in descending order when the passive controller discretely targets **a** $K = 8$, **b** $K = 16$, and **c** $K = 32$ randomly chosen nodes. Both controllers hold the same budget, $\mathscr{B} = N\langle d \rangle/60$. Allocations are coloured by the allocation group in **G** they belong to.

3 First-order Taylor expansion of the vote share for small allocations

Here, we provide the derivation of the Taylor expansion of the vote share (given by the heterogeneous mean-field approximation) for low allocations with respect to node degree, $(w_{ai} + w_{bi})/d_i \ll 1$. This result corresponds to equation (5) in the main manuscript.

We start from the HMF approximation of the total vote share at the steady-state X^{HMF} (equation (12) in the main manuscript):

$$X^{\text{HMF}} = \frac{1}{N}\left(\sum_i \frac{d_i}{d_i + w_{ai} + w_{bi}}\right)\left(\sum_i \frac{d_i w_{ai}}{d_i + w_{ai} + w_{bi}}\right)\left(\sum_i \frac{d_i(w_{ai} + w_{bi})}{d_i + w_{ai} + w_{bi}}\right)^{-1} + \frac{1}{N}\sum_i \frac{w_{ai}}{d_i + w_{ai} + w_{bi}}\,. \tag{1}$$

The optimisation of (1) under the budget constraint $\sum_i w_{ai} \leq \mathscr{B}_a$, can be written as

$$\max_{\boldsymbol{w}_a} X^{\mathrm{HMF}} + \frac{\lambda}{N}\left(\mathscr{B}_a - \sum_i w_{ai}\right) , \tag{2}$$

where λ is a Lagrange multiplier. As computing $\nabla_{\boldsymbol{w}_a} X^{\mathrm{HMF}} = \mathbf{0}$ leads to a system of polynomial equations for which no explicit solution can be computed, we derive a first-order approximation to the goal function in the limit of $\alpha_k = (w_{ai}+w_{bi})/d_i \to 0$. The new goal function appears as

$$X^{\mathrm{HMF}} = \frac{1}{N}\left(\sum_i \frac{1}{1+\alpha_i}\right)\left(\sum_i \frac{w_{ai}}{1+\alpha_i}\right)\left(\sum_i \frac{w_{ai}+w_{bi}}{1+\alpha_i}\right)^{-1} + \frac{1}{N}\sum_i \frac{\alpha_i}{1+\alpha_i}\frac{w_{ai}}{w_{ai}+w_{bi}} , \tag{3}$$

$$X^{\mathrm{HMF}} = X^{\mathrm{HMF}}\big|_{\alpha_i=0} + \sum_i \alpha_i \left.\frac{\partial X^{\mathrm{HM}}}{\partial \alpha_i}\right|_{\alpha_i=0} + \dots , \tag{4}$$

$$X^{\mathrm{HMF}} = \frac{\mathscr{B}_a}{\mathscr{B}_a+\mathscr{B}_b} + \frac{1}{N}\sum_i \frac{w_{ai}+w_{bi}}{d_i}\left(-\frac{\mathscr{B}_a}{\mathscr{B}_a+\mathscr{B}_b} + N\frac{-\mathscr{B}_b w_{ai}+\mathscr{B}_a w_{bi}}{(\mathscr{B}_a+\mathscr{B}_b)^2} + \frac{w_{ai}}{w_{ai}+w_{bi}}\right) + \dots \tag{5}$$

Solving $\partial\left[X^{\mathrm{HMF}} + \lambda/N\left(\sum_i w_{ai} - \mathscr{B}_a\right)\right]/\partial w_{ai} = 0$ leads to

$$w_{ai} = \frac{1}{2}\left(\frac{\mathscr{B}_a}{\mathscr{B}_b} - 1\right) w_{bi} + \frac{\mathscr{B}_a+\mathscr{B}_b}{2N} - \frac{(\mathscr{B}_a+\mathscr{B}_b)^2}{2N\mathscr{B}_b} d_i \lambda . \tag{6}$$

We can find the value of the Lagrange multiplier λ by replacing (6) in the constraint $\mathscr{B}_a = \sum_i w_{ai}$. Note that we also need to take into account the positivity constraint, $w_{ai} > 0$, so we must only consider the values of w_{ai} that stay above zero, leading to the final expression for w_{ai} as

$$w_{ai} = \frac{\mathscr{B}_a+\mathscr{B}_b}{2N} + \frac{1}{2}\left(\frac{\mathscr{B}_a}{\mathscr{B}_b} - 1\right)\left[w_{bi} + \frac{d_i}{\sum_{i:w_{ai}>0} d_i}\left(\mathscr{B}_b - \sum_{i:w_{ai}>0} w_{bi}\right)\right] . \tag{7}$$

Note that $w_{ai} > 0 \; \forall i \iff \sum_{i:w_{ai}>0} w_{bi} = \mathscr{B}_b \iff \lambda = 0 \iff (\mathscr{B}_b - \mathscr{B}_a) w_{bi} > \mathscr{B}_b (\mathscr{B}_a + \mathscr{B}_b)/N \; \forall w_{bi}$. Otherwise, it needs to be found which w_{ai} are breaking the positivity constraint. This can be done by iteratively setting all values that break the constraint to zero and readjusting the remaining values.

4 Zero-order Taylor expansion of the vote share for large allocations

Here, we also provide a derivation of the Taylor expansion of the vote share, but for high allocations with respect to node degree, $d_i/(w_{ai}+w_{bi}) \ll 1$. This result corresponds to equation (7) in the main manuscript.

We depart from the optimisation problem in (2). We derive a zero-order approximation to the goal function in the limit of $\alpha_i = d_i/(w_{ai}+w_{bi}) \to 0$. The new expression for X^{HMF} is then

$$X^{\mathrm{HMF}} = \frac{1}{N}\left(\sum_i \frac{\alpha_i}{1+\alpha_i}\right)\left(\sum_i \frac{d_i}{1+\alpha_i}\frac{w_{ai}}{w_{ai}+w_{bi}}\right)\left(\sum_i \frac{d_i}{1+\alpha_i}\right)^{-1} + \frac{1}{N}\sum_i \frac{1}{1+\alpha_i}\frac{w_{ai}}{w_{ai}+w_{bi}} , \tag{8}$$

$$X^{\mathrm{HMF}} = X^{\mathrm{HMF}}\big|_{\alpha_i=0} + \dots , \qquad X^{\mathrm{HMF}} = \frac{1}{N}\sum_i \frac{w_{ai}}{w_{ai}+w_{bi}} + \dots , \tag{9}$$

Solving $\partial\left[X^{\mathrm{HMF}} + \lambda/N\left(\sum_i w_{ai} - \mathscr{B}_a\right)\right]/\partial w_{ai} = 0$ leads to

$$w_{ai} = \sqrt{\frac{w_{bi}}{-\lambda}} - w_{bi} . \tag{10}$$

We can find the value of the Lagrange multiplier λ by solving its constraint $\mathscr{B}_a = \sum_i w_{ai}$. Note that we need to take into account the positivity constraint, $w_{ai} > 0$, so we must only consider the values that stay above zero, leading to the final expression for allocations w_{ai} as

$$w_{ai} = \left(\mathscr{B}_a + \sum_{k:w_{ak}>0} w_{bk} \right) \frac{\sqrt{w_{bi}}}{\sum_{k:w_{ak}>0} \sqrt{w_{bk}}} - w_{bi} \ . \tag{11}$$

Note that, again, it needs to be found which w_{ai} are breaking the positivity constraint by iteratively setting all values that break the constraint to zero and readjusting the remaining values.

5 Exploitability of a passive controller when deviating from uniform allocation

In the low-allocation limit of the HMF-approximation, the expression of the total vote share X^L given a passive controller with allocations $\boldsymbol{w}_b$ and an optimal active controller (equation (6) of the main manuscript) is

$$X^L = \frac{\mathscr{B}_a}{\mathscr{B}_a + \mathscr{B}_b} + \frac{\mathscr{B}_b}{4N^2} \sum_i \frac{(1 - w_{bi}/\langle \boldsymbol{w}_b \rangle)^2}{d_i} \ , \tag{12}$$

where $\langle \boldsymbol{w}_b \rangle = \mathscr{B}_b / N$.

To test for this effect, we explore a set of experiments where the passive controller targets nodes uniformly except for $n = 40$ perturbations of size $|1 - w_{bi}/\langle \boldsymbol{w}_b \rangle| = 1$ that are applied to nodes of a specific degree d_n. Fig 3 shows the deviations in vote share ΔX produced when such perturbations are applied. Analytical and numerical results match well, with deviations in vote share ΔX decreasing with the node degree of the perturbations as $\Delta X \propto d_n^{-1}$ as predicted in equation (12).

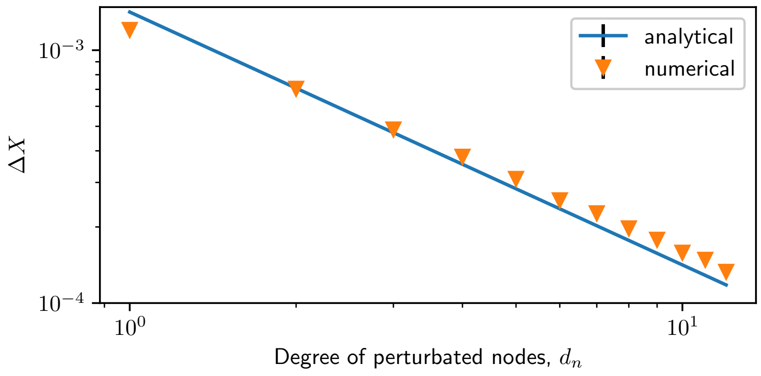

Figure 3. Increase in vote share ΔX for an optimal active controller when the passive controller deviates from uniform allocation on nodes with degree d_n with $n = 40$ perturbations of size $|1 - w_{bi}/\langle \boldsymbol{w}_b \rangle| = 1$. Error bars represent standard errors over 15 instances of the experiment and are smaller than the symbols. Both controllers hold the same budget $\mathscr{B} = N\langle d \rangle/60$.

6 Comparison of numerical and analytical results for optimal allocations in the continuous regime and budget disadvantage

This section complements the comparison of analytical and numerical results from Fig 2 of the main manuscript by analysing the case of budget disadvantage, $\mathscr{B}_a/\mathscr{B}_b = 0.1$. The goal of this comparisons is to verify the validity of the approximations made in the analytical results in the budget disadvantage scenario. As in Fig 2, we set the passive controller to target nodes with strengths w_{bi} randomly drawn from a uniform distribution. In Fig 4, we examine the dependence of optimal responses w_{ai} on allocations of the passive controller w_{bi} with allocations generally being smaller (left column), similar (middle column), or larger (right column) than node degree. In the figure, results corresponding to the HMF-approximation are given as curves, while numerical results are given as clouds of points. In the analytical results for the low-allocations limit (*solid lines*), the positivity constraints are active and the term that depends on d_i activates. This is particularly visible for low allocations (*left* panel), where we see different curves for each node degree.

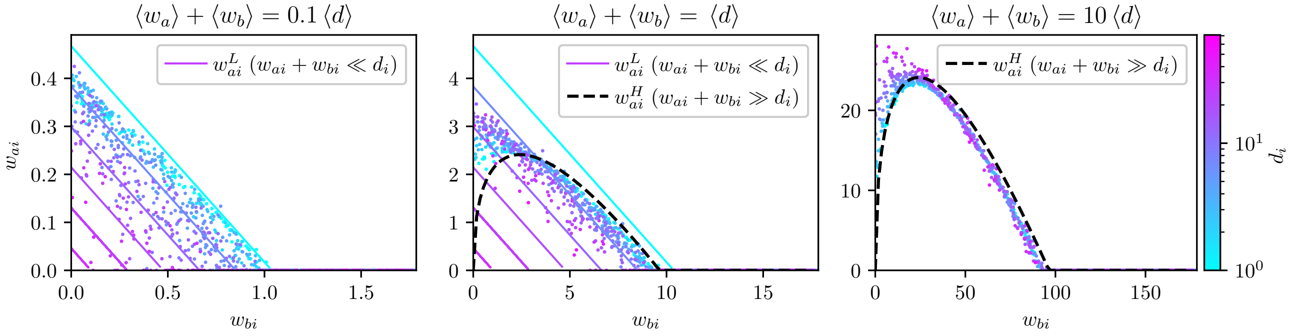

Figure 4. Comparison of analytical and numerical results for optimal influence allocations on the continuous regime. Dependence of optimal allocations w_{ai} on the allocations of a passive controller w_{bi} resolved by classes of nodes of different degrees, indicated by their colour. The active controller is in budget disadvantage, $\mathscr{B}_a/\mathscr{B}_b = 0.1$ and the passive controller targets continuously with weights randomly drawn from a uniform distribution. The value of external allocations (shown on top of each panel) are generally lower (*left*), equal (*middle*), or higher (*right*) than node degrees. Numerical results are given by a cloud of points coloured by node degree and analytical results are given by *curves* that correspond to equation (7) (*solid*) or equation (11) (*dashed*). Lines from w_{ai}^L are also coloured by node degree, while showing only one line every six degrees (i.e. for degrees 1,7,13,...).

7 Dependence of optimal influence allocations in the continuous regime on node degree

In this section, we directly show the relation between optimal allocations and node degree in the continuous regime. Fig 5**a** shows the dependence of optimal allocations on the weighted degree of nodes for the reference case of $K = 16$ random nodes targeted by the opponent. This scenario shows some degree of correlation between optimal influence allocations and node degree (with a Kendall rank coefficient of $\tau = 0.37$, as can be seen in Fig 5b of the main manuscript). In contrast, Fig 5**b** shows a similar scenario, but with the passive opponent not choosing her targets randomly but only targeting nodes whose neighbours have the lowest possible degree. We can see here that correlations are not as evident; the Kendall rank coefficient is $\tau = 0.05$, as can be seen in Fig 5c of the main manuscript. For instance, nodes whose weighted degree is above $d_i = 40$ receive very little allocation, and nodes with degree two, three and four receive higher allocation than most nodes with higher degree.

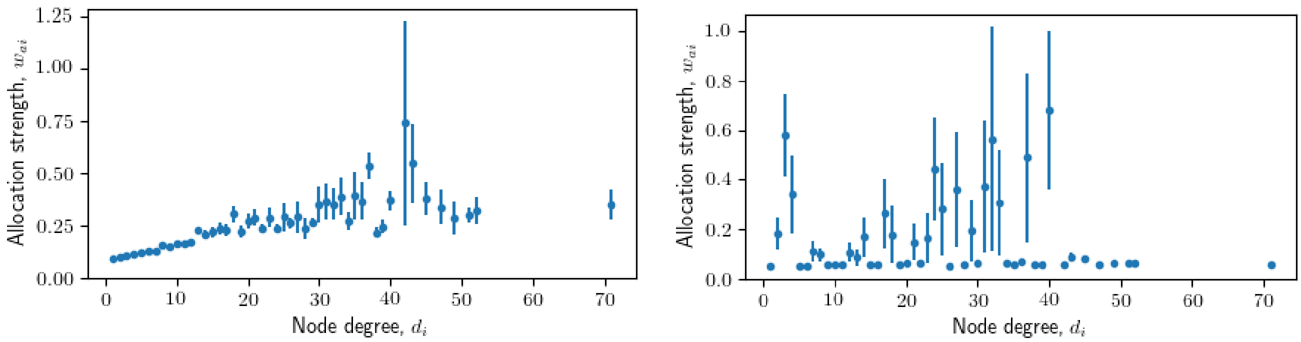

Figure 5. Dependence of optimal influence allocations w_ai in the continuous regime on node degree d_i. The passive controller targets $K = 16$ nodes in the network **a** randomly chosen or **b** whose neighbours have the lowest possible degree. Error bars represent standard errors of the mean (over all nodes with same degree). The total budget of both controllers sums up to $\mathscr{B}_a + \mathscr{B}_b = N\langle d \rangle/30$.

8 Shadowing, shielding and hub preferences in the discrete regime

In this section, we investigate the role that shadowing, shielding and node degree have in optimal allocations in the discrete regime against a passive controller who targets random nodes. We illustrate our results through numerical experiments for which we set both controllers to target K nodes in the network and vary K and the budget ratio $r = \mathscr{B}_a/\mathscr{B}_b$.

To test for the presence of shadowing, we measure the fraction ϕ of nodes targeted by the passive controller ($\mathbf{T}_b = \{i \,|\, w_{bi} = g\}$) that are also targeted by the active controller ($\mathbf{T}_a = \{i \,|\, w_{ai} = g\}$), $\phi = P(i \in \mathbf{T}_a \mid i \in \mathbf{T}_b)$. A strong presence of shadowing would be indicated by high values of ϕ, i.e. $\phi > 0.5$. We take as a reference the fraction ϕ_R obtained by an active controller who just targets random nodes in the network. Results strongly depend on the budget ratio of both controllers, as shown in Fig 6a. For equal budgets (*rhombi*), the active controller targets around 20% of the nodes targeted by the passive controller ($\phi \approx 0.2$) for most K. This is significantly different from the random behaviour for $K < 50$, with $\phi_R \approx 0$. So we can affirm that there is some presence of shadowing in the equal budget scenario, albeit very weak ($\phi = 0.2$). When in budget advantage (*squares*), shadowing is generally present with $\phi > 0.6$ for most K, so shielding is clearly present. In contrast, when in budget disadvantage (*circles*) the controller completely avoids targeting the nodes targeted by the passive controller, as $\phi \approx 0$ for all K. In conclusion, we see that optimal first-order strategies are qualitatively similar in the continuous and discrete regimes: with shadowing when in budget advantage, avoidance when in budget disadvantage, and a weak form of shadowing when in budget equality.

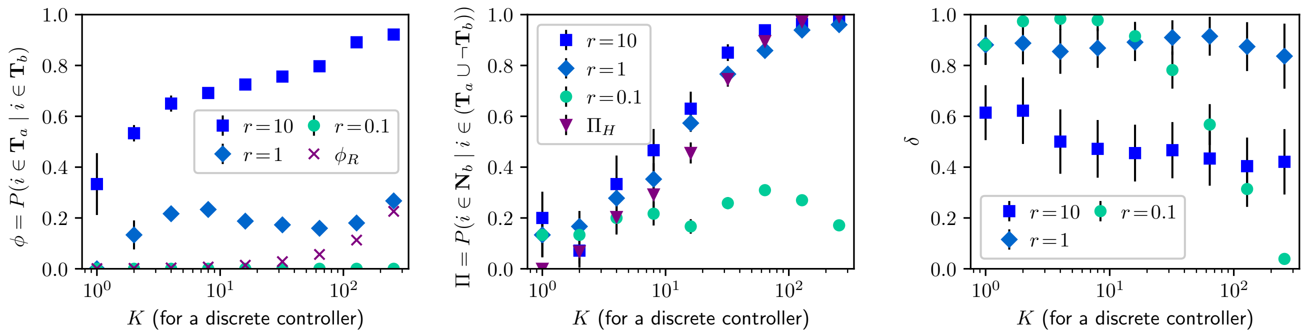

Figure 6. Amount of **a** shadowing, **b** shielding, and **c** degree dependence present in optimal discrete allocations for a range of numbers of nodes K targeted by either controller and different budget ratios $r = \mathscr{B}_a/\mathscr{B}_b$. Error bars represent standard errors over 15 instances of the experiments and the total budget of both controllers sums up to $\mathscr{B}_a + \mathscr{B}_b = N\langle d \rangle/30$.

To test for the presence of shielding in the discrete regime, we measure the probability Π that optimal allocations are directed towards neighbours of the nodes targeted by the passive controller, i.e. we measure $P(i \in \mathbf{N}_b \mid i \in (\mathbf{T}_a \cup \neg\mathbf{T}_b))$. Shielding would be evident with high values of Π. Note that we decouple the effect of shadowing in the probability by including the condition $i \in \neg\mathbf{T}_b$. Since shielding and node degree are related insofar as nodes with high degree are more likely to be in $\mathbf{N}_b$, we compare the results with what would be obtained by an active controller who targets the nodes with the highest degree in the network, labelled as Π_H. Results are again found to depend on the budget ratio r, as shown in Fig 6b. When in budget equality (*rhombi*) or superiority (*squares*), shielding-related probabilities Π steadily increase with K. However, these results are very similar to those obtained by the hub-targeting strategy Π_H so, inversely to what happens in the continuous regime, these values of Π likely are an artefact of a high-degree preference. Different to the other two scenarios, when in budget disadvantage (*circles*) shielding-related probabilities are below $\Pi < 0.4$ and largely unaffected by K. These low values of Π can be linked to an anti-shielding behaviour, where neighbours of the nodes targeted by the opponent tend to be avoided, as we showed in the main manuscript for the continuous regime (Fig 3b).

Last, we investigate the degree dependence of optimal allocations by looking at the mean degree of nodes targeted by the active controller $\langle d \rangle_{\mathbf{T}_a}$. We normalise these measurements via two reference strategies: targeting nodes with the highest (H) and lowest (L) degrees in the network. These reference strategies serve as upper and lower bounds on the obtainable average degree, respectively, with the normalised mean degree as $\delta = (\langle d \rangle_{\mathbf{T}_a} - \langle d \rangle_L)/(\langle d \rangle_H - \langle d \rangle_L)$. Based on the resulting δ, as seen in Fig 6c, we make three different observations depending on the budget ratio between controllers. First, when in budget equality (*rhombi*), the mean targeted degree stays above $\delta > 0.8$ irrespective of K, so the optimal strategy is greatly focused on targeting high-degree nodes. Second, when in budget disadvantage (*circles*), there is a transition in δ, as $\delta > 0.8$ for $K < 30$ and $\delta < 0.5$ for $K > 100$. This behaviour can be interpreted as an effect of anti-shielding: when K is low, hubs are targeted often since there is a preference towards hubs and they have low chances of belonging to $\mathbf{N}_b$. However, for large K, nodes with high degree almost surely belong to the group $\mathbf{N}_b$, which tend to be avoided, as seen in Fig 6b. Last, when in budget advantage (*squares*), mean targeted degrees are in the range $\delta \in (0.4, 0.65)$ for all K. The lower δ as compared to the budget equality scenario is a result of the preference towards high-degree nodes being partially discounted by the shadowing behaviour.

To summarise, we have seen the effect that the three proposed heuristics have in the discrete regime. First, related to degree dependence, optimal strategies in the discrete regime strongly favour targeting high-degree nodes in most scenarios.

This preference only gets discounted by shadowing when the active controller is in budget advantage, and by anti-shielding when in budget disadvantage if controllers target many nodes in the network. Second, the presence of first-order strategies is only significant when in budget inequality, leading to shadowing when in budget advantage and to avoidance when in budget disadvantage. Shielding is not present in any scenario of the discrete regime, although anti-shielding is present when in budget disadvantage.

9 Proof of uniqueness of Nash Equilibrium

We now demonstrate the uniqueness of our pure-strategy Nash Equilibrium solution. To prove this, we first show that our game belongs to the class of concave-convex games[2].

Let S be a subset of players from the player set P of a game, O the set of strategies chosen by the players, $u_i(X)$ the payoff player i obtains from the outcome O, and O^S the strategies chosen by the players in S. The game is concave-convex when the function $f(O) = \sum_{i \in S} u_i(O) - \sum_{i \in P \setminus S} u_i(O)$ is concave in O^S for each $O^{P \setminus S}$ and convex in $X^{P \setminus S}$ for each X^S. Moreover, it is a strictly concave-convex game if $u_i(O)$ is strictly concave in O_i. We derive function f for our two-person game as $f = u_A - u_B = 2X - 1$, with $u_A = X$ and $u_B = 1 - X$. We know that X (the vote share) is strictly concave in the strategy space $O_A = \boldsymbol{w}_a$ (refer to Section S10 for more details), hence f is also concave in O_A for any fixed $O_B = \boldsymbol{w}_b$. By symmetry, f is also strictly convex in $O_B = \boldsymbol{w}_b$ for any $O_A = \boldsymbol{w}_a$. The same can be shown using $f = u_B - u_A$, thus proving our game is a strictly concave-convex game.

Finally, we know that every strictly concave-convex game satisfies Rosen's diagonally strictly concave property[3], which asserts the existence of a unique pure-strategy Nash Equilibrium in such games[4].

10 Numerical results of an iterative approach to finding the Nash Equilibrium

This section shows numerical results for finding the pure-strategy Nash Equilibrium, which can be found by iteratively eliminating dominated strategies via gradient ascent until convergence. As can be seen in Fig 7, the normalised standard deviation of control allocations converges to zero (up to numerical precision). This Nash Equilibrium corresponds to both controllers targeting with equal strength all nodes in the network. This result is independent of the controllers' budgets, as it is seen in both panels of the figure. It is worth noting that the convergence of this iteration process is not guaranteed when budgets are not equal. In fact, it can be proven that in the low-budget limit, the process diverges if the budget ratio exceeds $\mathscr{B}_a/\mathscr{B}_b > 3 + 2\sqrt{2}$.

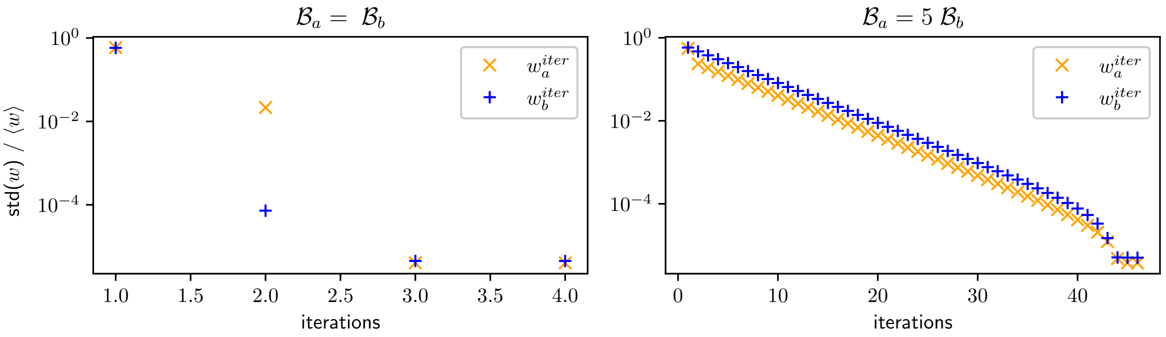

Figure 7. Changes in normalised standard deviation of control allocations of an A- controller and a B-controller when dominated strategies are iteratively removed for a setting of budget equality (left) and budget inequality (right). Initial allocations are randomly drawn from a uniform distribution and the total budget of both controllers sums up to $\mathscr{B}_a + \mathscr{B}_b = N\langle d \rangle/30$.

11 Extension to other network topologies

In this section, we have extended a selection of the experiments in the paper to other complex network topologies. We have chosen two synthetic networks —Barabasi-Albert networks[5] and scale-free networks built via configuration models[6] —and two real–world networks —a trust network[7] and a friendship network[8]. The Barabasi-Albert (BA) networks have been generated with each new node linking to five previous nodes. The scale-free networks have been generated via the configuration model[9] with an exponent of $\gamma = 3$ and setting the minimum number of connections per node to five. These parameters have been chosen

experimentally in such a way as to generate networks with similar connectivity level $\langle d \rangle$ as in the email interaction network from the main manuscript. Similarly, networks have been generated with the same number of nodes as the e-mail interaction network, namely $N = 1133$. Among the real–world networks, the online social network data corresponds to a weighted trust network among the users from the online social network *Advogato*[7,10]. The other real–world network corresponds to a self–reported friendship network between students in a high school in Marseilles, France, in December 2013[8]. For both real–world network, we have employed the biggest component of the network, ignored self–loops, and made the networks undirected. Table 1 contains summary statistics of all studied networks. Note that the Friendship network has a stronger assortativity (0.26) than the other networks (< 0.1).

Network	N	Mean degree	Max degree	Assortativity
Email interaction	1133	9.62	71	0.078
Barabasi-Albert	1133	9.96	129.2 ± 4.3	-0.051 ± 0.003
Scale-free	1133	9.22 ± 0.03	110.0 ± 1.0	-0.043 ± 0.003
Advogato	5054	13.13	747	-0.095
Friendship	128	6.27	17	0.260

Table 1. Statistics of the network employed on the main manuscript (Email interaction) and the networks employed here for the extension of experiments (Barabasi-Albert, Scale-free, Advogato, and Friendship).

In more detail, we have repeated the experiments in the main manuscript from Fig 1a —profile of optimal allocations—, Fig 1c —performance enhancement of using continuous allocations over discrete allocations—, Fig 4b —shadowing and shielding behaviours in the continuous regime—, Fig 5b —correlations between allocation strength and node degree—, and Fig 6—comparison of various heuristics to optimal allocations—.

Fig 8 shows the profile of optimal allocation distribution for each network topology. These figures are analogous to Fig 1a in the main manuscript. For the real–world networks Advogato and Friendship, the number K of nodes targeted by the opponent has been chosen as to preserve the percentage of nodes in the network targeted. Optimal allocations for the five studied network topologies share a very similar profile, with a smoother allocation profile visible for the Friendship network.

Fig 9 shows the percentage enhancement of control of continuous versus discrete optimal allocations for all network topologies studied here. These figures are analogous to Fig 1c in the main manuscript. The enhancement of control is generally similar for the five studied network topologies. A stronger effect can be seen in the Friendship network, where enhancement go up to 25% when budgets are equal and the opponent targets only one node in the network.

Fig 10 shows the average allocation strength given to nodes in the allocation groups related shadowing and shielding, $\mathbf{S} = \{\mathbf{T}_b, \mathbf{N}_b, \mathbf{R}\}$ for all network topologies studied. These figures are analogous to Fig 4b in the main manuscript. Shadowing and shielding generally have a similar effect in all of the studied networks, although shadowing is more and less present in the Advogato and Friendship networks, respectively. This effect could be related to the network size.

Fig 11 compares the Kendall rank correlation coefficients τ between allocation strength w_{ai} and node degree d_i between optimal allocations (*squares/triangles*) and allocations purely driven by a shielding heuristics (*circles*). These figures are analogous to Fig 5b in the main manuscript. The five network topologies show very similar behaviour regarding these correlations, both in optimal allocations and shielding heuristics.

Last, Figs 12 and 13 explore the effectiveness of different heuristics as compared to the optimised numerical results in the network topologies studied. The performance of each heuristic is captured by the difference in vote share ΔX between the heuristic and the numerically optimised numerical result. The smaller the gap in obtainable vote share ΔX between a heuristic and the numerically determined optimum, the stronger its contribution of the heuristic to optimal allocations. The figures illustrate results of the vote share gap for the three network topologies studied here, for three budget scenarios (advantage, equality, and disadvantage), and against a discrete passive controller who targets $K = 16$ random nodes in the network. Note that we make a clear separation between the discrete (*purple*) and continuous (*orange*) regimes and that their vote shares are compared to the discrete or continuous optimal strategies, respectively. The details of the implementation of each heuristic can be found in the *Methods* section of the main manuscript. These figures are analogous to Fig 6 in the main manuscript. Heuristics perform very similarly on the three network topologies from Fig 12. However, the network Friendship from Fig 13 shows a remarkably different behaviour in the discrete regime in that the degree–dependent heuristic does not perform significantly better than the other discrete heuristics.

12 Proof of concavity

We want to prove that the goal function $X = 1/N\, \mathbf{1}^T (L + W_a + W_b)^{-1} W_a \mathbf{1}$ is concave with respect to $\boldsymbol{w_a} \in \mathbb{R}^+$. This is important as it guarantees that the gradient ascent optimisation technique reaches the global maximum of the problem.

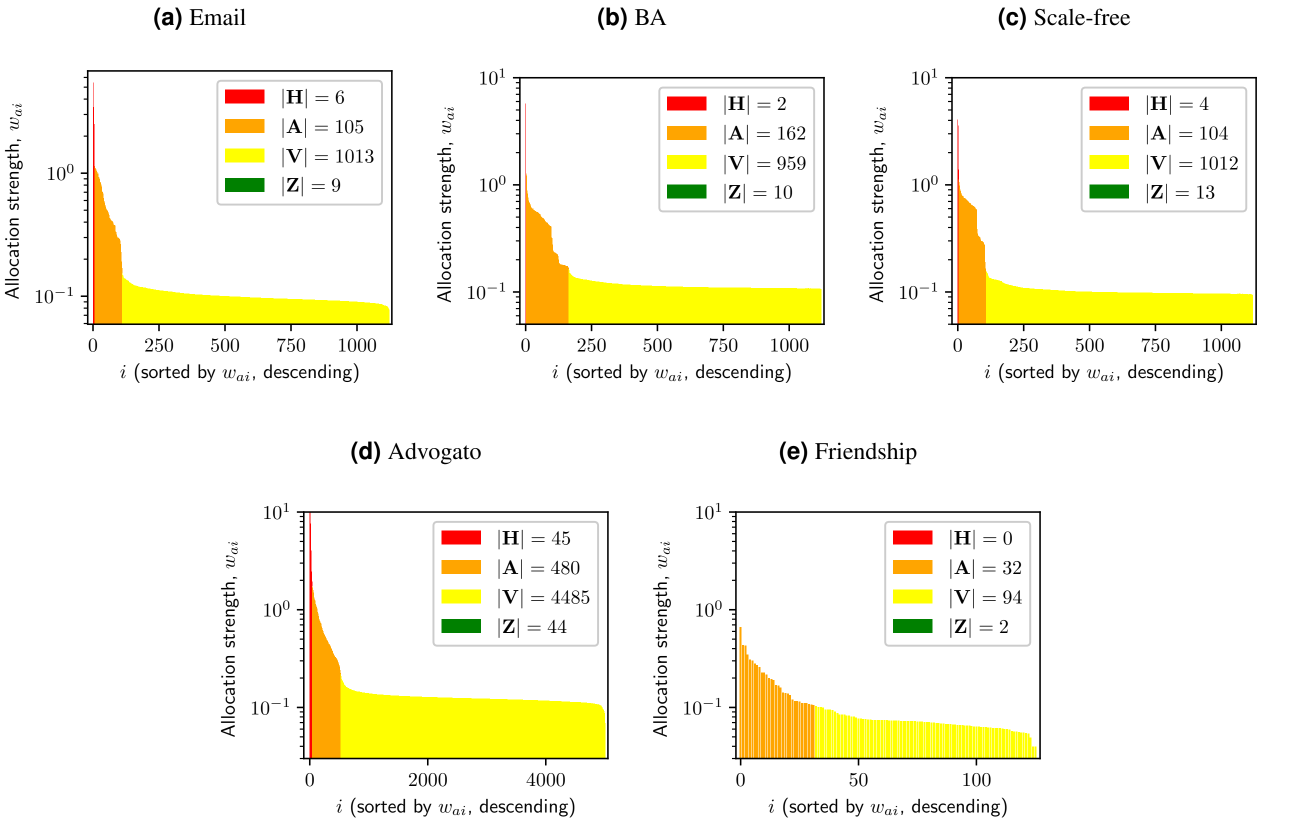

Figure 8. Distribution of optimal influence allocations for various network topologies. Allocations are sorted in descending order and the passive controller discretely targets around 1.5% of the nodes in the network (i.e., **a, b, c** $K = 16$, **d** $K = 71$, **e** $K = 2$), randomly chosen, and both controllers hold the same budget $\mathscr{B} = N\langle d_i \rangle / 60$. Allocations are coloured by the allocation group in **G** they belong to.

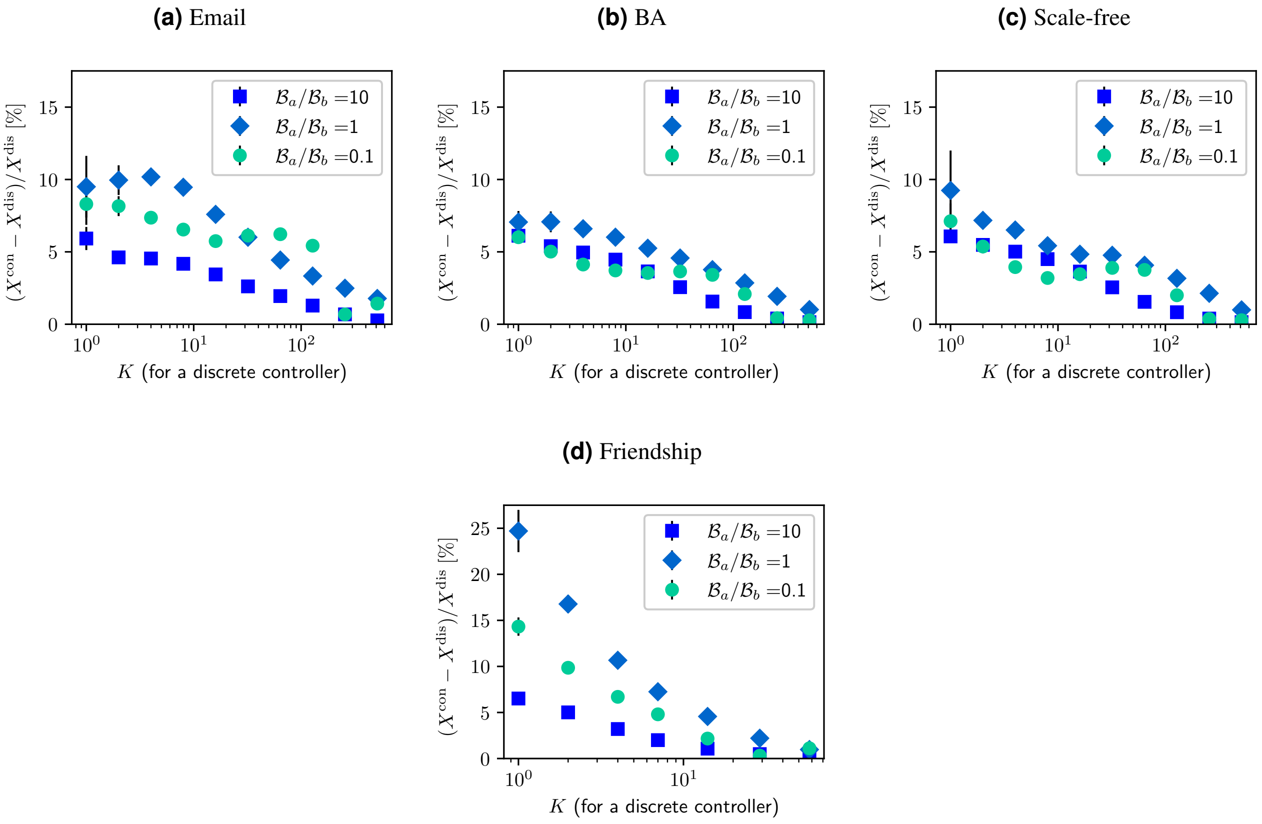

Figure 9. Percentage enhancement of control by optimal continuous allocations over optimal discrete allocations for various network topologies, with varying K (number of targeted nodes by discrete strategies) and budget ratios. Error bars represent standard errors over 15 experiment samples (5 in the case of **d**) and the total budget of both controllers sums up to $\mathscr{B}_a + \mathscr{B}_b = N\langle d \rangle/30$.

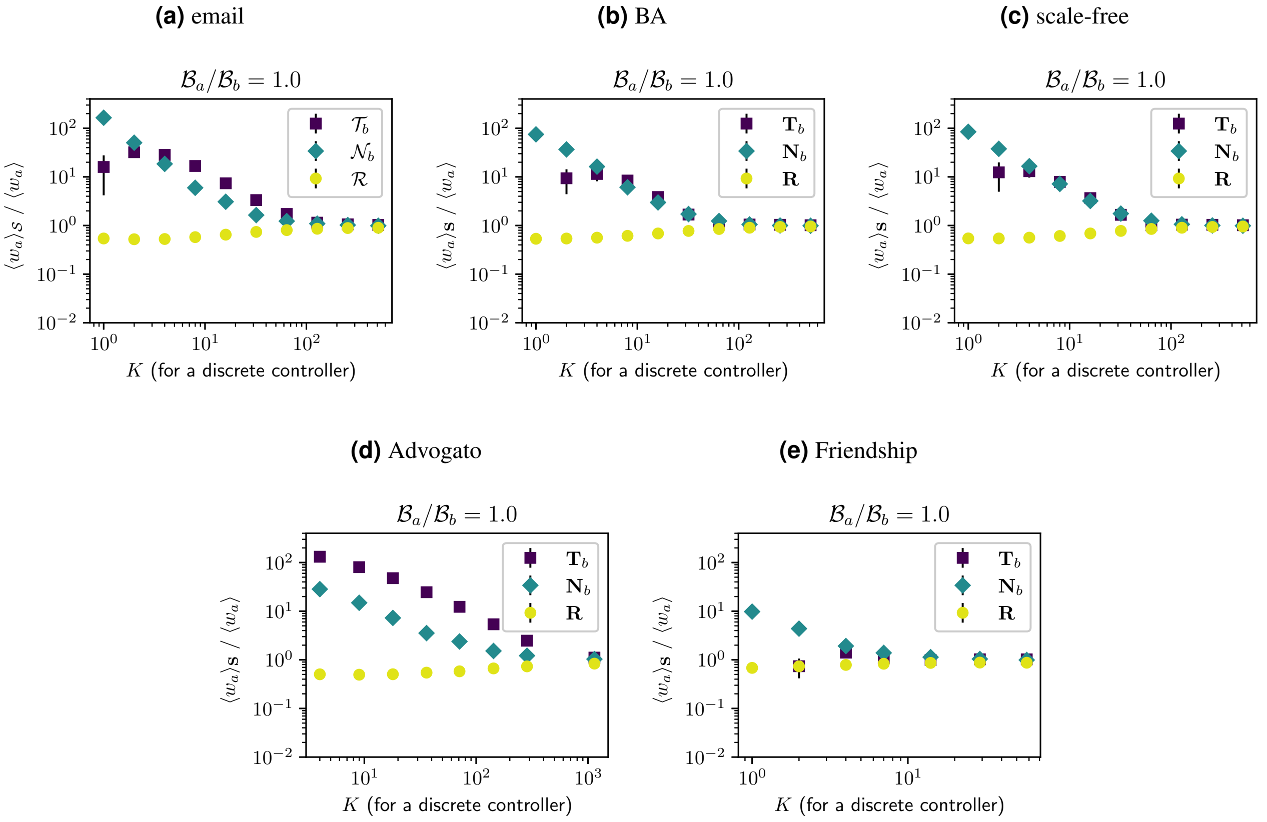

Figure 10. Average optimal allocations given to groups related to shadowing and shielding in various network topologies. Average allocations are normalised with respect to the total average allocation $\langle w_{ai} \rangle$ and both controllers hold equal budgets $\mathscr{B} = N\langle d \rangle/60.$. $\mathbf{T}_b$ is the set of nodes targeted by the passive opponent, $\mathbf{N}_b$ is the set of nodes that are neighbours of the nodes in $\mathbf{T}_b$, and $\mathbf{R}$ comprises the remaining nodes that do not belong to any of the other two groups. Error bars represent standard errors over 15 experiment samples (5 in the case of **d**).

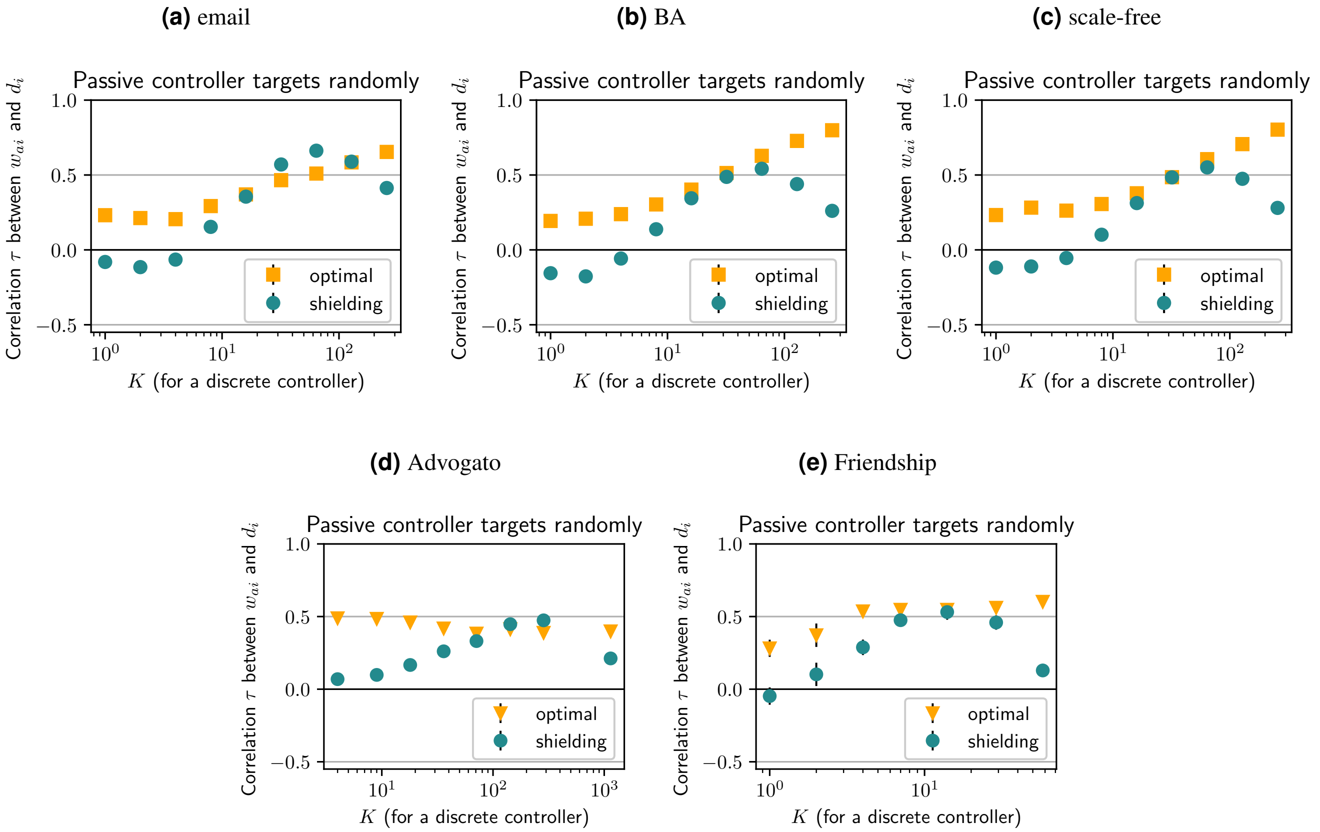

Figure 11. Kendall rank correlation coefficients τ_{wd} between allocation strength w_{ai} and node degree d_i in the continuous and discrete regimes and for various network topologies. The active controller targets nodes optimally (*squares*) or following a shielding strategy (*circles*). The passive controller targets random nodes in the network and the total budget of both controllers sums up to $\mathscr{B}_a + \mathscr{B}_b = N\langle d \rangle / 30$. Error bars represent standard errors over 15 experiment samples (5 in the case of **d**).

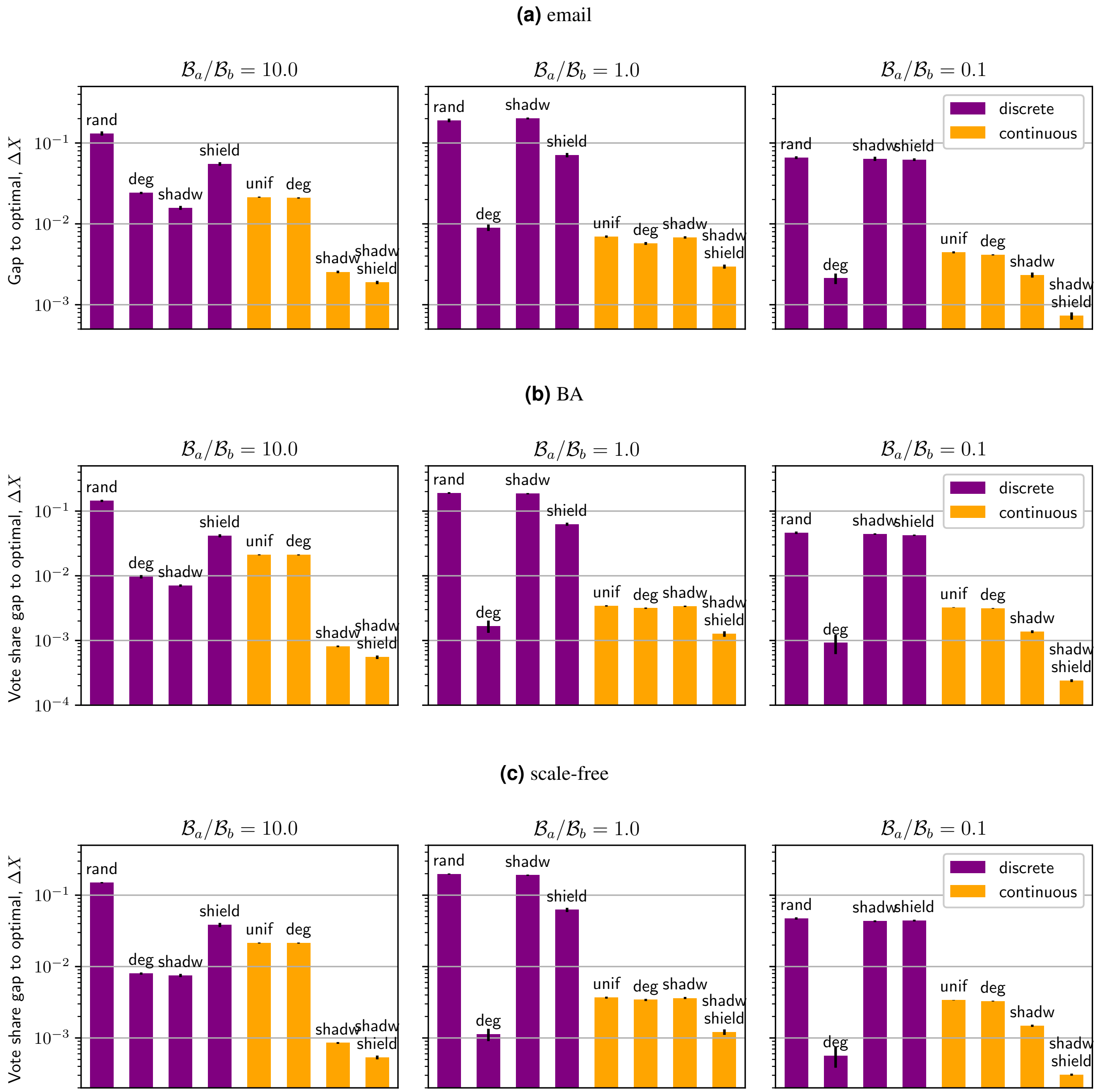

Figure 12. Comparison of various heuristics to optimal allocations. Bars represent the gap in vote share ΔX of the heuristics with respect to optimal numerical allocations for three different budget scenarios (as indicated on the top of the panels). Each bar represents one of the following heuristics: random (*rand*), degree-based (*deg*), shadowing-based (*shadw*), shielding-based (*shield*), uniform targeting (*unif*), combination of shadowing and shielding (*shadw shield*). The passive controller targets $K = 16$ random nodes in the network in a discrete fashion. Error bars represent standard errors for 15 instances of the experiments and the total budget of both controllers sums up to $\mathscr{B}_a + \mathscr{B}_b = N\langle d \rangle/30$.

(a) Friendship

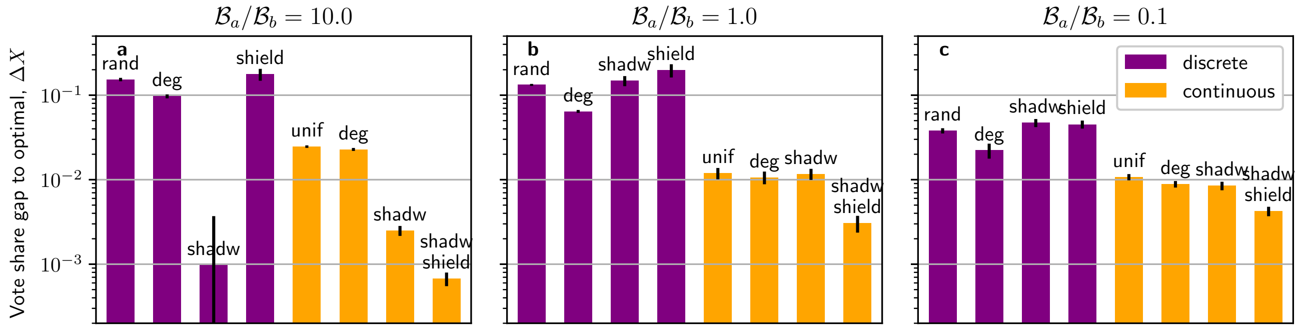

Figure 13. Comparison of various heuristics to optimal allocations. Bars represent the gap in vote share ΔX of the heuristics with respect to optimal numerical allocations for three different budget scenarios (as indicated on the top of the panels). Each bar represents one of the following heuristics: random (*rand*), degree-based (*deg*), shadowing-based (*shadw*), shielding-based (*shield*), uniform targeting (*unif*), combination of shadowing and shielding (*shadw shield*). The passive controller targets $K = 2$ random nodes in the network in a discrete fashion. Error bars represent standard errors for 15 instances of the experiments and the total budget of both controllers sums up to $\mathscr{B}_a + \mathscr{B}_b = N\langle d \rangle/30$.

We note that our model can be mapped to that of Abebe et al.[11], which considers opinion dynamics with innate opinions $s_i \in [0,1]$ and different susceptability of persuasion $\alpha_i \in [0,1]$ for each node, given by

$$x_i(t+1) = \alpha_i s_i + (1-\alpha_i)\frac{\sum_{j\in N(i)} x_j(t)}{deg(i)} , \tag{13}$$

where $N(i)$ is the neighbourhood of node i and $deg(i)$ is the degree of node i (d_i). This expression of the dynamics can be mapped to ours (equation (1) of the main manuscript) by applying the transformations $s_i = w_{ai}/(w_{ai}+w_{bi})$ and $\alpha_i = (w_{ai}+w_{bi})/(d_i+w_{ai}+w_{bi})$. Similar to our work, they maximise the vote share of opinions in the equilibrium by optimally adjusting α_i and prove the concavity of the goal function $Z = \mathbf{1}^T[I-(I-A)P]^{-1}A\mathbf{s}$ with respect to α_i, where A is a diagonal matrix with $A_{ii} = \alpha_i$, P is the random walk matrix $P = D^{-1}W$, and D is a diagonal matrix with $D_{ii} = d_i$. But Z is also equivalent to our goal function X, as

$$\begin{aligned} Z = \mathbf{1}^T[I-(I-A)P]^{-1}A\mathbf{s} = \mathbf{1}^T(I-[I-(W_a+W_b)(D+W_a+W_b)^{-1}]D^{-1}W)^{-1}(D+W_a+W_b)^{-1}W_a\mathbf{1} = \\ = \mathbf{1}^T(D+W_a+W_b-W)^{-1}W_a\mathbf{1} = \mathbf{1}^T(L+W_a+W_b)^{-1}W_a\mathbf{1} = NX \ . \end{aligned} \tag{14}$$

Since the mappings $s_i = w_{ai}/(w_{ai}+w_{bi})$ and $\alpha_i = (w_{ai}+w_{bi})/(d_i+w_{ai}+w_{bi})$ are monotonic, the proof of concavity from[11] can be applied to our problem by following analogous steps.

13 Iterations to optimal IM of the proposed heuristics

This section explores an alternative assessment of the proposed heuristics for IM. In Fig 6 of the main manuscript, we investigate gaps in vote share ΔX between each of the heuristics and the optimal strategy obtained numerically. Here, we provide an alternative assessment of the heuristics measuring computational costs to achieve similar quality solutions by performing numerical optimisation using gradient ascent. To achieve this we measure how many iterations of gradient-ascent are needed to reach vote shares X corresponding to each of the heuristics (Fig 14). That is, good heuristics correspond to large a number of iterations (i.e. high computational costs to achieve similar solutions) and poor heuristics to low numbers of iterations (or low computational costs to achieve similar outcomes by optimisation). From the figure, we can observe that all discrete strategies (*purple*) are already surpassed with less than 10 iterations of the gradient-ascent algorithm. In fact, in most cases the discrete *shadowing* and *shielding* strategies are already surpassed with a single iteration of the algorithm. Continuous heuristics (*orange*) are generally one order of magnitude better than discrete strategies (*purple*).

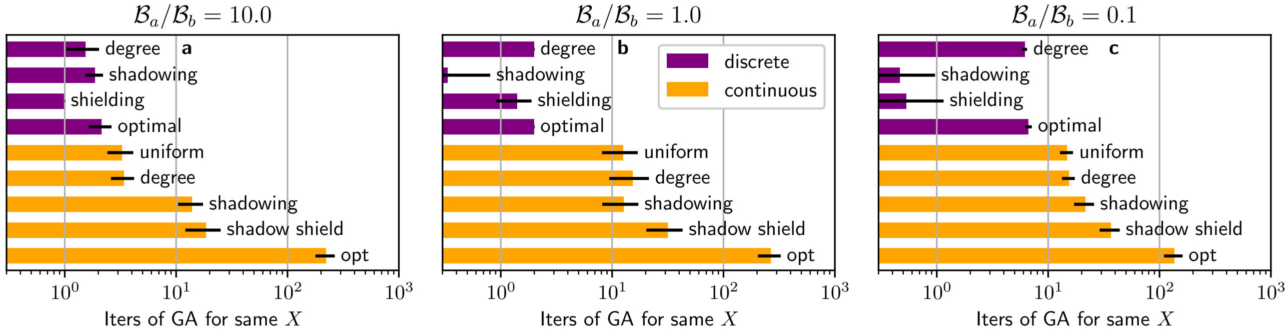

Figure 14. Alternative assessment of the proposed heuristics. On the x-axis, the number of iterations of the gradient-ascent algorithm required for obtaining a similar level of performance than the corresponding heuristic. On the y-axis, bars representing one of the following heuristics: degree-based (*degree*), shadowing-based (*shadowing*), shielding-based (*shielding*), uniform targeting (*uniform*), combination of shadowing and shielding (*shadow shield*). The passive controller randomly targets $K = 16$ nodes in the network in a discrete fashion and the total budget of both controllers sums up to $\mathscr{B}_a + \mathscr{B}_b = N\langle d \rangle/30$. Three budget scenarios are depicted (as indicated on the top of the panels). Error bars represent standard errors for 15 instances of the experiments. The initial allocation of the gradient-ascent algorithm is set at a discrete allocation on K random nodes.

References

1. Guimerà, R., Danon, L., Díaz-Guilera, A., Giralt, F. & Arenas, A. Self-similar community structure in a network of human interactions. *Phys. Rev. E* **68**, 065103, DOI: 10.1103/PhysRevE.68.065103 (2003).
2. Arrow, K. J. & Hurwicz, L. Stability of the gradient process in n-person games. *J. Soc. for Ind. Appl. Math.* **8**, 280–294 (1960).
3. Bervoets, S., Bravo, M. & Faure, M. Learning with minimal information in continuous games. *Theor. Econ.* **15**, 1471–1508 (2020).
4. Rosen, J. B. Existence and uniqueness of equilibrium points for concave n-person games. *Econom. J. Econom. Soc.* 520–534 (1965).
5. Dezső, Z. & Barabási, A.-L. Halting viruses in scale-free networks. *Phys. Rev. E* **65**, 055103, DOI: 10.1103/PhysRevE.65.055103 (2002).
6. Bayati, M., Kim, J. H. & Saberi, A. A Sequential Algorithm for Generating Random Graphs. *Algorithmica* **58**, 860–910, DOI: 10.1007/s00453-009-9340-1 (2010).
7. Massa, P., Salvetti, M. & Tomasoni, D. Bowling alone and trust decline in social network sites. In *Dependable, Autonomic and Secure Computing, 2009. DASC'09. Eighth IEEE International Conference on*, 658–663 (IEEE, 2009).
8. Mastrandrea, R., Fournet, J. & Barrat, A. Contact Patterns in a High School: A Comparison between Data Collected Using Wearable Sensors, Contact Diaries and Friendship Surveys. *PLOS ONE* **10**, e0136497, DOI: 10.1371/journal.pone.0136497 (2015).
9. Molloy, M. & Reed, B. A critical point for random graphs with a given degree sequence. *Random Struct. & Algorithms* **6**, 161–180, DOI: 10.1002/rsa.3240060204 (1995).
10. Rossi, R. A. & Ahmed, N. K. The network data repository with interactive graph analytics and visualization. In *AAAI* (2015).
11. Abebe, R., Kleinberg, J., Parkes, D. & Tsourakakis, C. E. Opinion Dynamics with Varying Susceptibility to Persuasion. In *Proceedings of the 24th ACM SIGKDD International Conference on Knowledge Discovery & Data Mining - KDD '18*, 1089–1098, DOI: 10.1145/3219819.3219983 (ACM Press, New York, New York, USA, 2018).
