# Supplementary material for: Shadowing and shielding: Effective heuristics for continuous influence maximisation in the voting dynamics
Source: PLoS One. 2021 Jun 18;16(6):e0252515. doi: 10.1371/journal.pone.0252515 (PMC8213046; doi:10.1371/journal.pone.0252515)
Supplement: Attachment — Submitted filename: Response to Reviewers.pdf [file pone.0252515.s003.pdf]

April 7, 2021

Guillermo Romero Moreno
School of Electronics and Computer Science
University of Southampton
Southampton SO171BJ
United Kingdom
Guillermo.RomeroMoreno@soton.ac.uk

Dear Hocine Cherifi,

Thank you for the opportunity to revise and resubmit our manuscript. We greatly appreciate the thoughtful and thorough feedback from the reviewers and we were excited to hear that our paper had the potential for publication.

We have now made significant revisions to the original manuscript —some are to the main text, but we also added results of additional experiments in the revised supplementary material. Most changes were made in the direction of improving the clarity of the text, rewriting sections where the previous text may have not been as clear as it should have or adding details that were lacking. We have also included new experiments in the supplementary material, including experiments on two other real–world networks. Below, you can find all the comments made by the reviewers, along with our point-by-point responses.

We believe the paper has improved because of the reviewers' feedback. We believe the revisions that we made address the reviewers' comments and we hope that the paper can be accepted for publication.

Thank you for your time and consideration.

Kind regards,

Guillermo Romero Moreno

We next include all the comments made by the reviewers, along with our replies. The first reviewer has raised the following points:

In this paper, the authors address the question of how to maximize influence in the context of the voter model with opinion leaders. I very much enjoyed reading the paper. It is clearly written, and the idea of integrating control theory with models of opinion dynamics is interesting and important. In particular, the demonstration that the 'shadowing' and 'shielding' strategies are two ends of a spectrum, which depends on the relative strength between the external influence weights and the node degree, is a nice result. My intuition, however, tells me that the optimal allocation for each node should closely correspond to the weighted degree of the node. The authors need to conduct further experiments to convince me otherwise.

We greatly appreciate the high engagement of the reviewer and the detail of the feedback provided.

The intuition of the reviewer is right, there is some correlation between influence allocations and the weighted degree of nodes. This relation we explore and discuss in the subsection named *Hub preferences and dependence on node degree* (line 336). There —and as can be seen in Fig 5— we show that some correlation generally exists between allocation strength and the weighted degree. But, importantly, we also show that this correlation is greatly related to a shielding strategy, as nodes with high degrees are more likely to be neighbours to those targeted by the opponent. In the paper, we provide two strong arguments in support of this. First, we show that rank correlations between strength allocations and weighted degree are weak for scenarios where the opponent targets only a few nodes (i.e. for low K). The reason is that, in such scenarios, the nodes targeted by the opponent do not have enough neighbours for making the correlations visible. Second, we show a contrived scenario where the opponent only targets nodes whose neighbours have the lowest possible degree. In such cases, correlations between influence allocations and weighted degrees completely vanish, pointing to shielding as the main cause for the presence of such correlations in other scenarios.

I have included below several comments that need to be addressed by the authors:

*** Comment 1. The authors need to provide additional details regarding the experiments in Figure 1. What is the size of the network? How are the weights (i.e., wij) determined in this experiment?**

We have modified the introducing paragraph from the *Results* section (line 138) to specify that the size of the network is $N = 1133$ and that the weights are determined from a real–world network that we take as a sample —the email–interaction network released by Guimèra et al.

*** Comment 2. Regarding the optimal control allocation (not 'shadowing' and 'shielding'), the authors need to include a plot relating the weighted degrees of nodes (i.e., di) to their corresponding optimal allocations (i.e., wai). What do you get?**

As we have argued above, we explore rank correlations between optimal allocations and weighted degrees in the subsection *Hub preferences and dependence on node degree.* We are happy to include the plots that the reviewer is requesting in the supplementary materials and also include them below for your convenience.

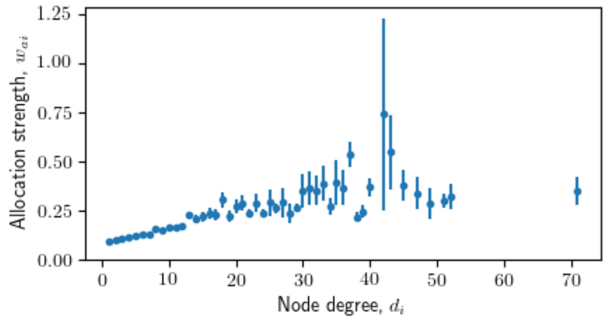

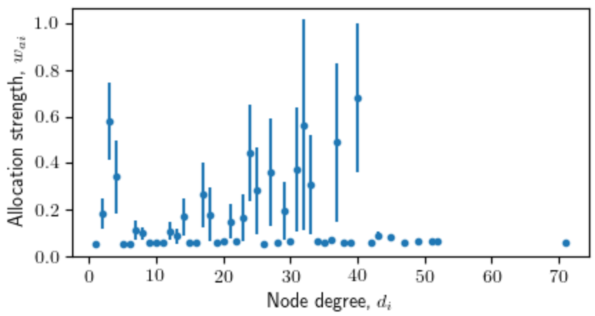

The plot on the left shows the dependence of optimal allocations on the weighted degree of nodes for the reference case of $K = 16$ random nodes targeted by the opponent and with equal budgets. Bars represent standard errors of the mean (over nodes with equal degree). This scenario indeed shows some degree of correlation, with a Kendall rank coefficient of $\tau = 0.37$, as can be seen in Fig 5b of the main manuscript. In contrast, the plot on the right shows a similar scenario, but with the passive opponent not choosing her targets randomly but only targeting nodes whose neighbours have the lowest possible degree. We can see here that correlations are not as evident; the Kendall rank coefficient is $\tau = 0.05$, as can be seen in Fig 5c of the main manuscript. For instance, nodes whose weighted degree is above $d_i = 40$ receive a very weak allocation, and nodes with degree two, three and four receive higher allocation than most nodes with higher degree.

*** Comment 3. What is the distribution of the optimal allocation values (Figure 1)? When changing the model's parameters (K, B, wij, network structure), do you see any interesting scaling regimes related to the optimal allocation distribution? How does the allocation distribution compare with the weighted degree distribution?**

We did explore the allocation distribution for other values of K before choosing the final setting for Fig 1. We are happy to also include some of them in the supplementary material. We also include these extra figures below, which compare allocation distributions for an opponent targeting $K = 8$ (left), $K = 16$ (middle), and $K = 32$ (right) nodes.

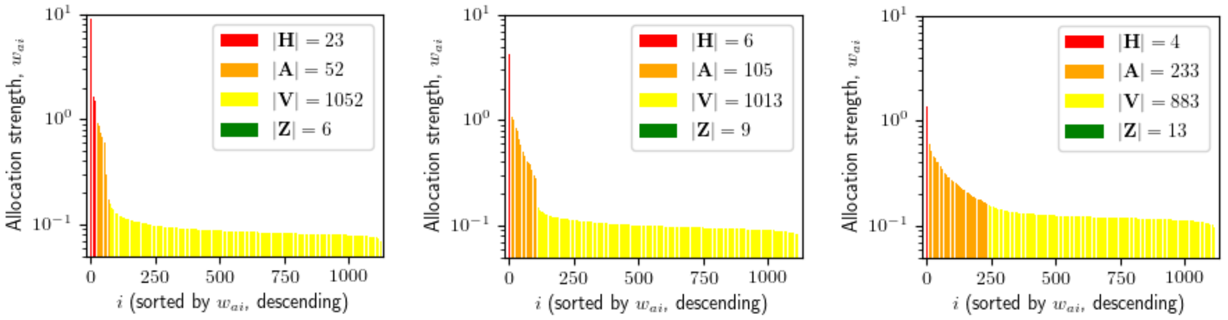

As can be seen from the figures, the allocation distribution smoothens as K increases. In Fig 1b of the main manuscript, we have indirectly captured this phenomenon by showing the entropy of the distribution decreasing with K.

For a direct comparison, we include below the distribution of weighted node degrees in a plot with identical structure as the ones above depicting the distribution of optimal allocations. There are significant differences between the distributions, as the weighted resembles a power–law, while in optimal allocations differences between the few nodes with the largest allocation and those with low allocation are much bigger.

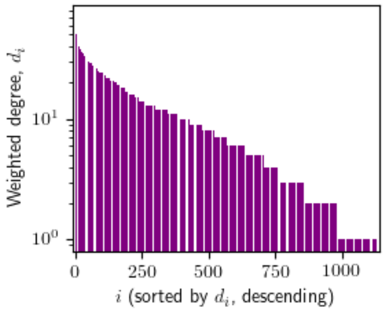

Regarding other network structures (and, consequently, other values for w_{ij}), we include an extension of the experiments to other network topologies in the supplementary material (Section 11). In particular, we explore results in two synthetically created topologies —a Barabasi–Albert network and a network with a scale–free distribution— and two real–world networks. Allocation profiles do not differ considerably in these other network topologies.

*** Comment 4. In your model you assume that the control vector is independent of time. In real life scenarios (e.g. in the context of political campaigns) it is likely that campaigners change their allocation based on real-time information on the current vote share of nodes in the system (e.g. as estimated by polls). It would be nice to comment on this possible extension of your model.**

We find this suggestion very valuable and we have consequently included a statement related to a time-dependent modification of the model. This can be found in lines 532–535 of the revised manuscript, in the *Discussion* section.

*** Comment 5. Perhaps I am missing something, but don't you have an unneeded "N" in your equation of the entropy (on Page 5)?**

The expression $logN$ is used as a pre-factor to the computation of the entropy for normalising entropies to the interval $[0, 1]$. We have clarified this in the revised version of the manuscript (line 183).

*** Comment 6. The following paper, I believe, is one of the first papers that introduced the concept of "zealots" in the context of opinion dynamics (along with Mobilia's paper): Chinellato, D. D., de Aguiar, M. A., Epstein, I. R., Braha, D., & Bar-Yam, Y. (2007). Dynamical response of networks under external perturbations: exact results. arXiv preprint arXiv:0705.4607. An extension of this paper also appeared in: Chinellato, D. D., Epstein, I. R., Braha, D., Bar-Yam, Y., & de Aguiar, M. A. (2015). Dynamical response of networks under external perturbations: exact results. Journal of Statistical Physics, 159(2), 221-230. It would be appropriate to include them in your references.**

We have included the suggested references in the *Introduction*, line 36.

The remarks from the second reviewer, along with our replies, are as follows.

The authors investigate the problem of influence maximization in presence of a competitor in the network. Also, they relax the constraint of discrete budget allocation, which is one of the most used assumptions in settings considered for the influence maximization problem. Specifically, the authors study the problem when different amounts may be allocated to different nodes, i.e., continuous influence maximization. First, they analyze the case when the competitor is passive, i.e., it has already influenced some of the nodes. Next, they also analyze two active opponents, using a game theoretical approach. Their results suggest that while degree-based methods are better suited for discrete budget allocations, in continuous influence maximization, mimicking the targets of the opponent or selecting the direct neighbors of the opponent's targets are strategies superior to selecting hub nodes. The paper is well written, and includes interesting results obtained with a sound methodology. Based on these considerations, I recommend the publication of the paper provided that the authors address the following issues.

We are thankful to the reviewer for the thoughtfulness with which they engaged the paper and the detail of suggestions, which we have addressed below.

[major]

1) Is it fair to make a straight comparison of performance between degree centrality and other optimization strategies? When using shadowing or shielding, the heuristics have the information on the moves of the opponent. However, when using the degree heuristic, such an information is not available to the method. This fact creates an obvious disadvantage for degree centrality. The observation does not aim at diminishing the importance of the results of the paper. However, the very fact that the information available to the methods is different should be clearly pointed out.

We thank the reviewer for highlighting this fact, which we agree that it was not sufficiently stressed in the previous version of the manuscript. We have added a paragraph and a table to the revised manuscript explaining in detail the different levels of information required for each heuristic. They can be found at the end of the *Heuristics* subsection (lines 639–644).

We would also like to clarify here that our aim is not to find a best–performing heuristic but explore to what extent optimal allocations can be described by each heuristic. In fact, the heuristics related to the continuous regime are hierarchical: simpler heuristics are sub-cases of heuristics higher up in the rank and therefore are by definition unable to beat the higher classes. For such cases, a lower heuristic that obtains similar performance than a higher one is already *beating* them, in the sense that they are achieving similar performance but with a leaner information requirement.

We hope that the new additions to the *Heuristics* subsection will make these points clearer.

2) Why haven't the authors tried their methods on any other real networks? Additional tests, especially on networks with different size, are needed to understand the extent of the results of the paper.

We have performed additional tests on two other real–world networks with different sizes, as suggested by the reviewer. Results for these tests can be found in the supplementary material, Section 11. All results hold for these other networks, as well as they do in other synthetically created networks, as shown in Section 11 of the supplementary material.

3) Results are presented as they would be valid for very generic settings. However, it should be clearly stated that the results of the paper are valid for the competitive setting only. In particular, the disadvantages of the degree heuristic should be made more apparent than they are in the current version of the paper. Also, it should be remarked that the presented results do not extend to the case where only a single entity is responsible for network spreading.

We have now included a few lines in the revised version of the manuscript discussing this concern (in the *Discussion* section, lines 485–491). There, we emphasise that our results mainly refer to a competitive scenario with two opposing controllers. However, as we have also argued in the newly added lines, the competitive scenario can be fully mapped to a case of influence maximisation with a single external controller and a population that exhibits resistance to being influenced, so there is at least one case of a single entity responsible for network spreading in which our results are fully applicable. In this case, the resistance from members of the population is equivalent to a passive controller that targets nodes with a strength proportional to their degree, and hence a degree–dependent heuristic can be very effective for this scenario.

[minor]

4) Line 161: Where did the constant 8 come from? Is it an arbitrary value selected on the basis of the information on the system available to the authors? If so, are there any other constants that make sense, and how do these constant values affect the results of node groupings?

The constant 8 is indeed arbitrarily chosen and based on an exploration of sensible partitions of the allocation distribution displayed in Fig 1. Indeed, there is a range of other values that would be acceptable for our purposes. Also, there are other equivalent ways to define the same partitioning, such as dividing allocations into fixed ranges of strength (not depending on the mean value) or by ranking. However, we do not consider that this constant plays an important role, as results related to this arbitrary grouping are mainly illustrative and their goal is to gain intuition on the allocation distribution. Such results are always followed by others performed more systematically, dispensing with this particular node grouping.

We have made a small amend to the introduction of the partitioning to further clarify this point (line 165).

5) Fig4: What does it happen when the active controller has a budget advantage? Also, in Fig.4c why aren't any points for N_b in the range x=3 to x=65? Is there an intuitive explanation for this behavior?

The case of budget advantage is very similar to that of budget equality (just with higher allocations to nodes within T_b) and hence we decided not to include it due to its lack of interest. We have added a line in the revised version of the manuscript (line 341) clarifying this.

Regarding the absence of some points for N_b in Fig 4c, the reason is that those points have a value of zero, and they cannot appear in the figure as its y-axis is logarithmic. We have added a note in the figure caption explaining this.

6) Ref. 43: The name of the first author should be Erkol Ş.

The bibliography entry has been fixed, showing now the name of the author correctly.
